# Ehrlichia chaffeensis Uses an Invasin To Suppress Reactive Oxygen Species Generation by Macrophages via CD147-Dependent Inhibition of Vav1 To Block Rac1 Activation

**DOI:** 10.1128/mBio.00267-20

**Published:** 2020-04-21

**Authors:** Omid Teymournejad, Yasuko Rikihisa

**Affiliations:** a Department of Veterinary Biosciences, The Ohio State University, Columbus, Ohio, USA; University of Texas Medical Branch; Brigham and Women’s Hospital

**Keywords:** CD147, *Ehrlichia chaffeensis*, invasin, Rac1, Vav1, macrophages, reactive oxygen species

## Abstract

The obligatory intracellular pathogen Ehrlichia chaffeensis lacks most factors that could respond to oxidative stress (a host cell defense mechanism). We previously found that the C terminus of *Ehrlichia* surface invasin, entry-triggering protein of *Ehrlichia* (EtpE; EtpE-C) directly binds mammalian DNase X, a glycosylphosphatidylinositol-anchored cell surface receptor and that binding is required to induce bacterial entry and simultaneously to block the generation of reactive oxygen species (ROS) by host monocytes and macrophages. However, how the EtpE-C−DNase X complex mediates the ROS blockade was unknown. A mammalian transmembrane glycoprotein CD147 (basigin) binds to the EtpE-DNase X complex and is required for *Ehrlichia* entry and infection of host cells. Here, we found that bone marrow-derived macrophages (BMDM) from myeloid cell lineage-selective CD147-null mice had significantly reduced *Ehrlichia*-induced or EtpE-C-induced blockade of ROS generation in response to phorbol myristate acetate. In BMDM from CD147-null mice, nucleofection with CD147 partially restored the *Ehrlichia*-mediated inhibition of ROS generation. Indeed, CD147-null mice as well as their BMDM were resistant to *Ehrlichia* infection. Moreover, in human monocytes, anti-CD147 partially abrogated EtpE-C-induced blockade of ROS generation. Both *Ehrlichia* and EtpE-C could block activation of the small GTPase Rac1 (which in turn activates phagocyte NADPH oxidase) and suppress activation of Vav1, a hematopoietic-specific Rho/Rac guanine nucleotide exchange factor by phorbol myristate acetate. Vav1 suppression by *Ehrlichia* was CD147 dependent. E. chaffeensis is the first example of pathogens that block Rac1 activation to colonize macrophages. Furthermore, *Ehrlichia* uses EtpE to hijack the unique host DNase X-CD147-Vav1 signaling to block Rac1 activation.

## Abstract (precis)

Ehrlichia chaffeensis is an obligatory intracellular bacterium with the capability of causing an emerging infectious disease called human monocytic ehrlichiosis. *E. chaffeensis* preferentially infects monocytes and macrophages, professional phagocytes, equipped with an arsenal of antimicrobial mechanisms, including rapid reactive oxygen species (ROS) generation upon encountering bacteria. As *Ehrlichia* isolated from host cells are readily killed upon exposure to ROS, *Ehrlichia* must have evolved a unique mechanism to safely enter phagocytes. We discovered that binding of the *Ehrlichia* surface invasin to the host cell surface receptor not only triggers *Ehrlichia* entry but also blocks ROS generation by the host cells by mobilizing a novel intracellular signaling pathway. Knowledge of the mechanisms by which ROS production is inhibited may lead to the development of therapeutics for ehrlichiosis as well as other ROS-related pathologies.

## INTRODUCTION

Ehrlichia chaffeensis is an obligatory intracellular bacterium. To infect host monocytes and macrophages, E. chaffeensis uses the C terminus of its unique outer membrane invasin, entry-triggering protein of *Ehrlichia* (EtpE; EtpE-C), to directly bind the host cell DNase X, a cell surface glycosylphosphatidylinositol-anchored receptor. This binding drives *E. chaffeensis* entry by engaging the type I transmembrane glycoprotein CD147 (basigin/extracellular matrix metalloproteinase inducer) and cytoplasmic heterogeneous nuclear ribonucleoprotein K (hnRNPK), which leads to the neuronal Wiskott-Aldrich syndrome protein (N-WASP)-dependent polymerization of actin (1). Phagocytes, such as monocytes and neutrophils, produce NADPH oxidase, a multicomponent enzyme composed of a heterodimeric cytochrome *b*_558_ integral membrane component (gp91*^phox^* [NOX2] and p22*^phox^*), three cytoplasmic subunits (p67*^phox^*, p47*^phox^*, and p40*^phox^*), and the small GTPase Rac1 or Rac2 (2). In resting phagocytes, the NADPH oxidase components are dissociated, and hence, the enzyme is inactive. Phagocyte-activating agents such as phorbol myristate acetate (PMA), invading pathogens, or *N*-formyl peptide (3) can induce rapid assembly of all components of NOX2 into a holoenzyme to catalyze the production of superoxide anion (O_2_^–^) from molecular oxygen. O_2_^–^ serves as the starting material for the production of powerful microbicidal reactive oxygen species (ROS), including hydrogen peroxide (H_2_O_2_), oxidized halogens, hydroxyl radicals, and singlet oxygen (4). Paradoxically, *E. chaffeensis* isolated from host cells is quite sensitive to ROS, and infectivity decreases rapidly once the bacterium is exposed to ROS (5). In fact, the *E. chaffeensis* genome lacks genes encoding enzymes that facilitate ROS detoxification, free radical scavenging, repair of ROS-induced damage, and the oxidative stress response (5, 6). Therefore, our previous studies have addressed whether *E. chaffeensis* can inhibit the activation of NADPH oxidase in phagocytes. Our previous work demonstrated that *E. chaffeensis* does not induce ROS production in human monocytes and rapidly blocks O_2_^–^ generation induced by a powerful stimulus, namely, PMA. This inhibition is specific to monocytes (*E. chaffeensis* cannot block ROS production in neutrophils), and a host cell surface protein is required (5). Recently, we identified DNase X as the host cell surface protein required for this block of ROS production, which is initiated by the binding of *E. chaffeensis* EtpE-C to DNase X (7). However, the mechanism by which DNase X mediates blockade of NADPH oxidase activation was unknown. Because EtpE-C binding to DNase X also triggers *E. chaffeensis* entry into host cells, we investigated downstream signaling related to the ROS blockade. DNase X receptor-dependent entry of *E. chaffeensis* and of recombinant EtpE-C (rEtpE-C)-coated beads into mammalian host cells requires actin polymerization and activation of an actin nucleation-promoting factor, N-WASP (1). Our recent study revealed that N-WASP activation is not involved in the inhibition of ROS production initiated by *E. chaffeensis* or EtpE-C (7). In the present study, we investigated whether CD147, which is recruited to DNase X upon EtpE-C binding to DNase X (1), is required for inhibiting ROS production. Toward this goal, we developed myeloid cell lineage-selective CD147-null mice.

Activated Rac GTPases are required for signaling cascades that lead to the activation of NADPH oxidase and are initiated by binding of *N*-formylmethionyl-leucyl-phenylalanine or other receptor agonists to the plasma membranes of neutrophils and monocytes (8–11). There are two isoforms of Rac, namely, Rac1 and Rac2, and Rac2 is the predominant isoform in human neutrophils, whereas Rac1 predominates in monocytes, accounting for 90% of cellular Rac (11). GDP-bound Rac is inactive and primarily found in the cytoplasm as a complex with a Rac-specific GDP dissociation inhibitor (12), whereas GTP-bound Rac is active and localizes to the plasma membrane (13). For Rac activation, GTP-for-GDP exchange is facilitated by a membrane-localized, Rac-specific guanine nucleotide exchange factor (13), and Rac becomes inactivated upon GTP hydrolysis catalyzed by a Rac-specific GTPase-activating protein (14). Vav1 is a hemopoiesis-specific Rho/Rac guanine nucleotide exchange factor that plays a prominent role in adhesion-mediated suppression of ROS generation in neutrophils (15). Therefore, we investigated whether Rac1 and Vav1 are involved in *E. chaffeensis*-induced or EtpE-C-induced suppression of ROS generation in human monocytes. Our findings provide important molecular insights into how an obligatory intracellular pathogen may subvert NADPH oxidase-related signaling during its entry to facilitate subsequent colonization of phagocytes.

## RESULTS

### Suppression of ROS generation by *E. chaffeensis* is dependent on CD147.

**FIG 1:**
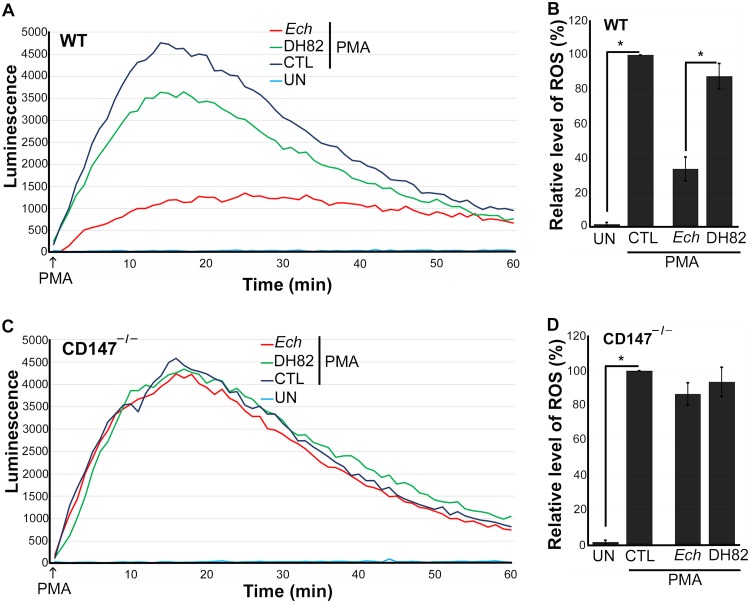
*E. chaffeensis* blocks PMA-induced ROS generation by WT BMDM but not by CD147^–/–^ BMDM. BMDM from WT mice (A and B) or CD147^–/–^ mice (C and D) were preincubated with luminol in HBSSd for 15 min and then incubated with *E. chaffeensis* (*Ech*) isolated from infected DH82 cells, DH82 cell lysate, or HBSSd (control [CTL]) at 37°C for 30 min. ROS generation was continuously recorded as the relative chemiluminescence of oxidized luminol after the addition of PMA (200 nM, indicated by arrows) (A and C). UN, unstimulated BMDM in HBSSd without PMA addition. The area under the curve was measured over 60 min after PMA addition and is shown relative to ROS generation in the control with PMA, which was considered 100% in panels B and D. Results represent the means plus standard deviations (SD) (error bars) from at least three independent experiments and were compared by Student’s *t* test. Values that are significantly different (*P* < 0.05) are indicated by a bar and asterisk.

**FIG 2:**
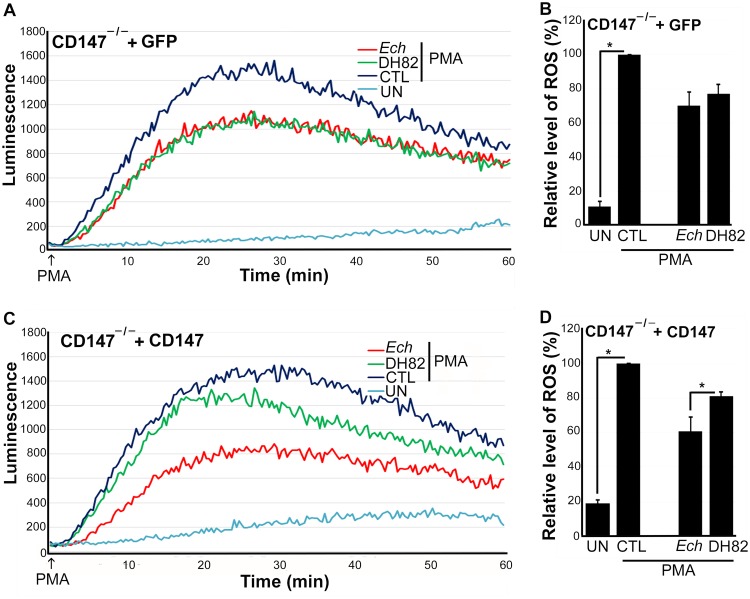
Nucleofection with CD147, but not control GFP plasmid, complements BMDM from CD147^–/–^ mice for suppression of ROS generation by *E. chaffeensis*. BMDM from CD147^–/–^ mice were nucleofected with a CD147-encoding plasmid (CD147) or control plasmid for 1 day (A). Nucleofected cells were preincubated with luminol in HBSSd for 15 min and then incubated with *E. chaffeensis* (*Ech*), DH82 cell lysate, or HBSSd (control [CTL]) at 37°C for 30 min. ROS generation was induced with PMA, recorded (A and C), and analyzed (B and D). UN, unstimulated BMDM in HBSSd without PMA addition. Results represent the means plus SD from at least three independent experiments and were compared by Student’s *t* test. *, *P* < 0.05.

Mammalian DNase X is a glycosylphosphatidylinositol-anchored, cell surface receptor. Upon *E. chaffeensis* binding to DNase X, the transmembrane protein CD147 is recruited to the EtpE-C−DNase X complex, which results in a relay of the extracellular signal (i.e., *E. chaffeensis* binding) to the cytoplasm to trigger actin polymerization (1). Hence, we examined whether CD147 also inhibits ROS generation in macrophages in response to *E. chaffeensis* (7). Knockout of *CD147* (*Bsg*, encoding basigin) in mice results in spermatocyte apoptosis, germ cell degeneration, and infertility (16), and consequently, CD147 knockout (CD147^–/–^) mice are difficult to obtain. Thus, to test the requirement for CD147 in *E. chaffeensis*-induced or EtpE-C-induced suppression of ROS generation, we generated a conditional null allele by introducing two loxP sites flanking exons 2 to 8 of *CD147* (*Bsg*). *Bsg^flox/flox^* pups were born at the expected Mendelian ratio, with a growth rate similar to that of wild-type (WT) mice. After crossing these mice with Lyz2-Cre (lysozyme promoter-driven Cre recombinase) transgenic mice, CD147 expression was specifically inactivated in myelocytic cells in the resulting *bsg^flox/flox-lyz2-Cre^* mice. The birth and growth rates of *bsg^flox/flox-lyz2-Cre^* mice were similar to those of WT mice. Using *bsg^flox/flox-lyz2-Cre^* mice, we examined whether CD147 is required for *E. chaffeensis*-induced suppression of ROS generation with a luminol-dependent chemiluminescence assay, which measures total (intra- and extracellular) O_2_^–^ and H_2_O_2_ production using luminol, a small membrane-permeable luminogenic molecule (17). We measured levels of ROS generated in response to PMA using WT and *bsg^flox/flox-lyz2-Cre^* mouse bone marrow-derived macrophages (BMDM) preincubated for 30 min with isolated *E. chaffeensis* or with lysate of canine macrophage DH82 cells (used as a negative control because *E. chaffeensis* was cultured in DH82 cells, and consequently, there is carryover of host cell proteins in *E. chaffeensis* bacteria isolated from these cells). Similar to results obtained with human peripheral blood-derived macrophages (5) and mouse BMDM (7), mouse BMDM generated copious ROS upon PMA treatment (Fig. 1A and B). Similar results were obtained with CD147^–/–^ BMDM, indicating that CD147 does not directly modulate PMA-induced ROS generation (Fig. 1C and D). Preincubation of WT BMDM with *E. chaffeensis* for 30 min significantly blocked PMA-induced ROS generation. Unlike WT BMDM, however, preincubation of CD147^–/–^ BMDM with *E. chaffeensis* for 30 min did not block PMA-induced ROS generation (Fig. 1C and D), indicating that CD147 mediates the inhibition of PMA-induced ROS generation by *E. chaffeensis*. With WT BMDM, there was slight diminution of ROS production by DH82 cell lysates treated with PMA compared with PMA alone, which was likely attributable to the nonenzymic and enzymatic antioxidants present in the DH82 cell lysates but not in CD147^–/–^ BMDM, suggesting a more general role for CD147 in mediating suppression of ROS generation by monocytes. The inability of *E. chaffeensis* to induce suppression of ROS generation in CD147^–/–^ BMDM was partially restored by nucleofection with a plasmid encoding full-length CD147 but not one encoding green fluorescent protein (GFP) (control; Fig. 2) (18), suggesting that CD147 is required for suppression of ROS generation. Nucleofection of BMDM with plasmid, however, reduced their response to PMA with respect to ROS production based on the overall luminescence values between Fig. 1 and 2. Taken together, these results indicated that CD147 is primarily responsible for mediating *E. chaffeensis*-induced suppression of ROS generation in BMDM.

### EtpE-C blocks ROS production by WT BMDM but not CD147^–/–^ BMDM.

**FIG 3:**
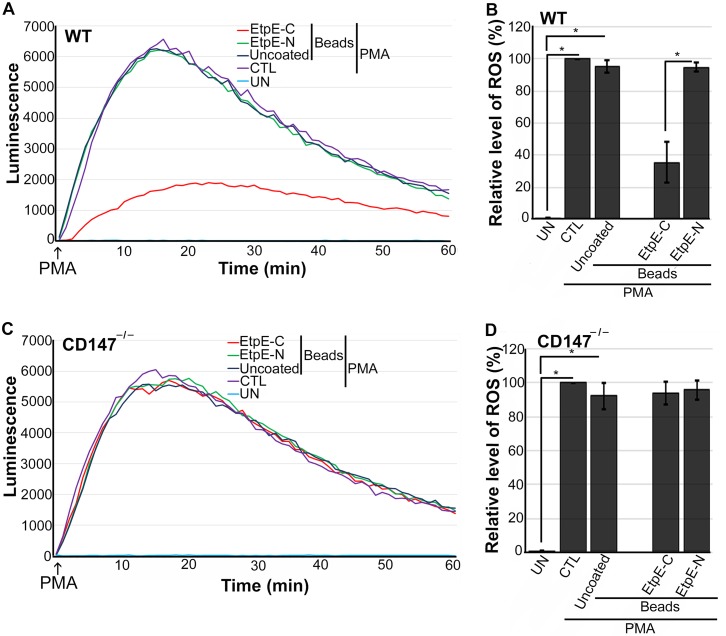
EtpE-C-coated beads block PMA-induced ROS generation by WT BMDM but not CD147^–/–^ BMDM. BMDM from WT mice (A and B) or CD147^–/–^ mice (C and D) were preincubated with luminol in HBSSd for 15 min and then incubated at 37°C for 30 min with beads coated with 40 ng of EtpE-C or EtpE-N or with uncoated beads or with HBSSd (control [CTL]). ROS generation was induced with PMA, recorded (A and C), and analyzed (B and D), and the results are presented as in Fig. 2. UN, unstimulated BMDM in HBSSd without PMA addition. Results represent the means plus SD from at least three independent experiments and were compared by Student’s *t* test. *, *P* < 0.05.

EtpE-C directly binds DNase X to trigger *E. chaffeensis* entry into macrophages (19). Previous work demonstrated that inert latex beads (of a size similar to that of an *E. chaffeensis* bacterium) that are either uncoated or coated with the N-terminal portion of EtpE (as a recombinant protein, rEtpE-N) are phagocytosed by BMDM independently of DNase X, but beads that are coated with rEtpE-C are endocytosed in a DNase X-dependent manner (19). Therefore, we examined the effects of EtpE-C-coated beads on CD147-dependent suppression of ROS generation; rEtpE-N-coated beads and uncoated beads were used as negative controls. Uncoated or rEtpE-N-coated beads did not inhibit ROS generation in WT or CD147^–/–^ BMDM in response to PMA (Fig. 3A to D). However, rEtpE-C-coated beads significantly reduced ROS generation by WT BMDM but not by CD147^–/–^ BMDM (Fig. 3A to D). These results suggested that CD147 mediates EtpE-C- and DNase X complex-induced suppression of ROS generation in BMDM.

### CD147^–/–^ BMDM and mice are resistant to infection with *E. chaffeensis*.

**FIG 4:**
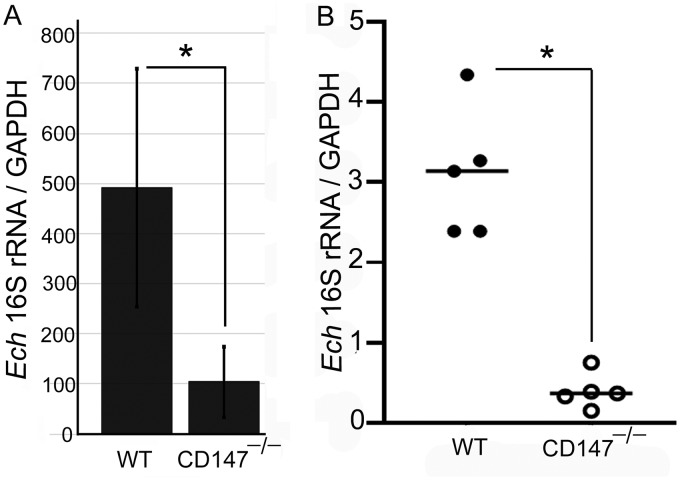
CD147^–/–^ BMDM and mice are resistant to infection with *E. chaffeensis*. BMDM from WT or CD147^–/–^ mice were incubated with *E. chaffeensis* for 48 h (A). WT and CD147^–/–^ mice (five mice per group) were infected with *E. chaffeensis* for 4 days (B). Quantitative PCR was performed for the *E. chaffeensis* (*Ech*) 16S rRNA gene and mouse GAPDH to compare infection levels in BMDM and blood specimens. Results represent the means plus SD from at least three independent experiments and were compared by Student’s *t* test. *, *P* < 0.05.

Given that CD147^–/–^ BMDM did not block ROS generation in response to PMA and that CD147 is required for *E. chaffeensis* entry into host cells (1), we compared *E. chaffeensis* load in CD147^–/–^ BMDM in culture at 2 days postinfection and in peripheral blood of mice at 4 days postinfection. In CD147^–/–^ BMDM and mice, there was significantly less infection than in WT BMDM and mice (Fig. 4), indicating that effective *in vitro* and *in vivo* infection of *E. chaffeensis* requires CD147.

### CD147 is required for suppression of ROS generation by EtpE-C in human peripheral blood monocytes.

**FIG 5:**
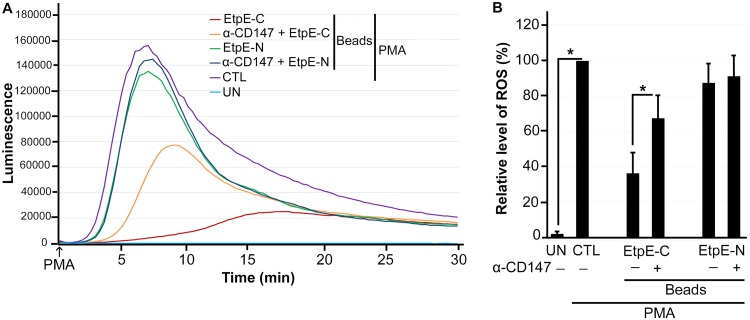
EtpE-C-coated beads do not block PMA-induced ROS generation in human monocytes pretreated with anti-CD147. Human monocytes were preincubated with luminol with or without 10 μg/ml anti-CD147 (α-CD147) for 30 min at 37°C and then incubated with beads coated with 40 ng of EtpE-C or EtpE-N (∼5 × 10^6^ beads) or with HBSSd (control [CTL]) at 37°C for 30 min. ROS generation was continuously recorded as the relative chemiluminescence of oxidized luminol after the addition of PMA (200 nM, indicated by arrows) (A). UN, unstimulated human monocytes in HBSSd without PMA addition. The area under the curve was measured over 30 min after PMA addition and is shown relative to ROS generation in the control with PMA, which was considered 100% in panel B. Results represent the means plus SD from at least three independent experiments and were compared by Student’s *t* test. *, *P* < 0.05.

A monoclonal antibody against CD147 (MEM-M6/6), which targets the membrane-proximal Ig1 domain of CD147, blocks DNase X-mediated *E. chaffeensis* entry by inhibiting signaling that leads to intracellular actin polymerization (1). To replicate the results obtained with mouse CD147^–/–^ BMDM (Fig. 3) in human macrophages, human peripheral blood monocytes pretreated with or without MEM-M6/6 were incubated with rEtpE-C-coated or rEtpE-N-coated beads and then stimulated with PMA. MEM-M6/6 also significantly reduced the suppression of ROS generation by EtpE-C (Fig. 5); EtpE-N-coated beads had no effect, with or without MEM-M6/6 (Fig. 5). These results indicated that suppression of ROS generation by EtpE-C is mediated by CD147 in human monocytes.

### Rac1 activation by PMA is blocked upon binding of *E. chaffeensis* or rEtpE-C-coated beads to human monocytes.

**FIG 6:**
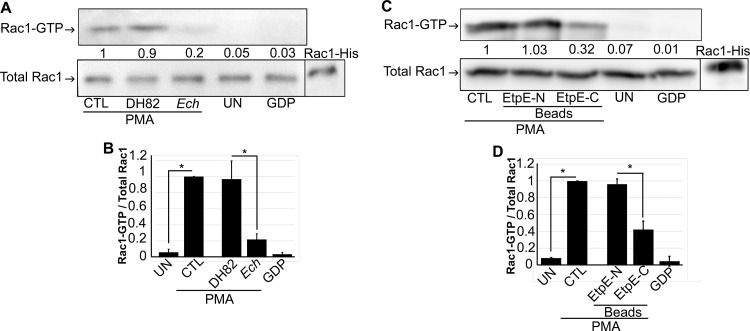
*E. chaffeensis* and EtpE-C inhibit PMA-induced Rac1 activation in human monocytes. (A) Human peripheral blood monocytes were preincubated with *E. chaffeensis* (*Ech*), DH82 cell lysate (DH82), or HBSSd (control [CTL]) for 1 h, followed by stimulation with PMA (200 nM) for 5 min. (B) Human peripheral blood monocytes were preincubated with EtpE-N- or EtpE-C-coated beads or HBSSd (CTL) for 30 min, followed by stimulation with PMA (200 nM) for 5 min. UN, unstimulated monocytes in HBSSd without PMA addition. Rac1-GTP was pulled down and measured along with total Rac1 by Western blotting. GDP, lysate of monocytes loaded with GDP. Rac1-His, recombinant Rac1-His. The relative amounts of Rac1-GTP were calculated by normalizing the band intensities to that of total Rac1, and the ratio observed in the CTL (HBSSd + PMA) was arbitrarily set at 1. Results are presented as the means ± SD from at least three independent experiments and were compared by analysis of variance. *, *P* < 0.05.

The GTPase Rac1 is a molecular switch for NADPH oxidase complex assembly and activation in human monocytes (11). Hence, we examined whether binding of *E. chaffeensis* to human monocytes inhibits subsequent Rac1 activation (Rac1-GTP) in response to PMA. When human peripheral blood monocytes were preincubated with *E. chaffeensis* or DH82 cell lysate for 30 min and then stimulated with PMA, *E. chaffeensis*, but not DH82 cell lysate, significantly blocked Rac1 activation in response to PMA (Fig. 6A and B). When human peripheral blood monocytes were preincubated with rEtpE-C-coated, rEtpE-N-coated, or uncoated beads for 30 min and then stimulated with PMA, the EtpE-C-coated beads, but not the EtpE-N-coated beads, significantly blocked Rac1 activation (Fig. 6C and D). Hence, suppression of ROS generation in response to PMA by *E. chaffeensis* or EtpE-C is likely due to inhibition of Rac1 activation.

### Vav1 activation by PMA is blocked upon binding of *E. chaffeensis* or rEtpE-C-coated beads to human monocytes.

**FIG 7:**
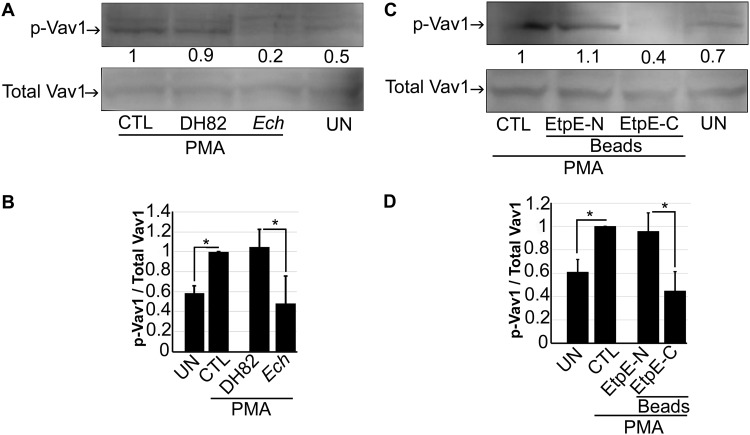
*E. chaffeensis* and EtpE-C inhibit PMA-induced Vav1 activation in THP-1 cells. (A) THP-1 cells were preincubated with *E. chaffeensis* (*Ech*), DH82 cell lysate, or HBSSd (control [CTL]) for 30 min, followed by stimulation with PMA (200 nM) for 5 min. (B) THP-1 cells were preincubated with EtpE-N- or EtpE-C-coated beads or HBSSd CTL for 30 min, followed by stimulation with PMA (200 nM) for 5 min. UN, unstimulated monocytes in HBSSd without PMA addition. Native Vav1 was pulled down, and phosphorylated Vav1 (p-Vav1) was detected with anti-phosphotyrosine, and total Vav1 was detected with anti-Vav1 by Western blotting. The relative amounts of p-Vav1 were calculated by normalizing the band intensities with that of total Vav1, and the ratio observed in CTL (HBSSd + PMA) was arbitrarily set at 1. Results are presented as the means plus SD from at least three independent experiments and were compared by analysis of variance. *, *P* < 0.05.

Vav proteins are GDP/GTP exchange factors for Rho/Rac GTPases, and tyrosine phosphorylation activates Vav function (20–23). Vav1 is exclusively expressed in hematopoietic cells and plays an important role in the activation of hematopoietic cells, including monocytes and macrophages (24). Given that PMA activates Rac1 in human monocytes, we examined whether Vav1 is activated (tyrosine phosphorylated) in human monocytes in response to PMA. As shown in Fig. 7A and B, PMA treatment activated endogenous Vav1 in human monocytes (THP-1 cells); THP-1 cells were used because, in this assay, the amount of endogenous Vav1 is below the limit of detection in human monocytes derived from even ∼4 × 10^7^ peripheral blood mononuclear leukocytes (PBML). *E. chaffeensis* and EtpE-C inhibited Vav1 activation in THP-1 cells in response to PMA (Fig. 7A to D). Hence, *E. chaffeensis* and EtpE-C binding to DNase X on the surfaces of human peripheral blood monocytes inhibits ROS generation in response to PMA via inhibition of the Vav1-Rac1 axis.

### Inhibition of Vav1 activation by *E. chaffeensis* is mediated by CD147.

**FIG 8:**
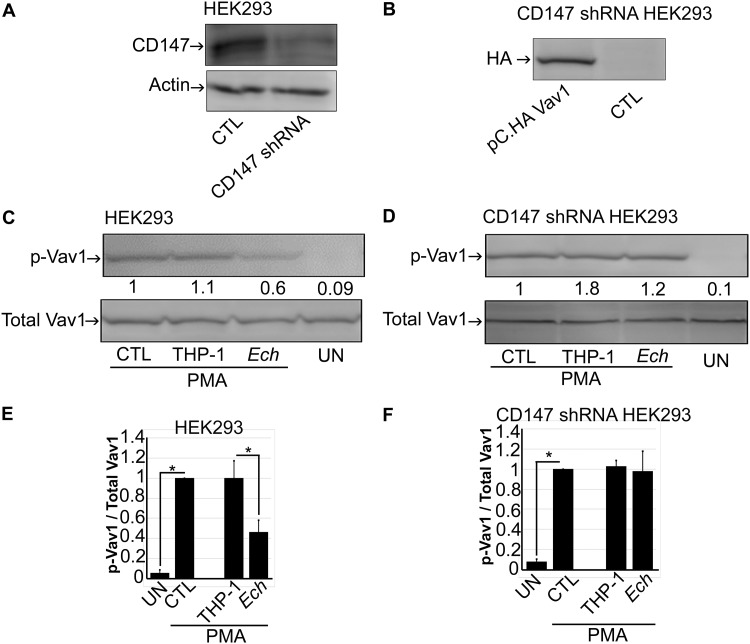
Inhibition of Vav1 activation by *E. chaffeensis* is mediated by CD147. (A) Western blotting for CD147 in HEK293 cells (CTL) and CD147 shRNA-transformed HEK293 cells. (B) Western blotting for HA-Vav1 in pC.HA Vav1-transfected or nontransfected (CTL) CD147 shRNA-transformed HEK293 cells using anti-HA. (C and D) pC.HA Vav1-transfected HEK293 cells (C) and pC.HA Vav1-transfected CD147 shRNA-transformed HEK293 cells (D) were preincubated with *E. chaffeensis* (*Ech*), THP-1 cell lysate (THP-1), or HBSSd (control [CTL]) for 30 min, followed by stimulation with PMA (200 nM) for 5 min. Total Vav1 and p-Vav1 were detected with Western blotting. The relative amounts of p-Vav1 were calculated by normalizing the band intensities with that of total Vav1, and the ratio observed in CTL (HBSSd + PMA) was arbitrarily set at 1. Results are presented as the means plus SD from at least three independent experiments and were compared by analysis of variance. *, *P* < 0.05.

Given that CD147 mediates suppression of ROS generation by *E. chaffeensis* and that *E. chaffeensis* suppresses Vav1 activation, we examined whether inhibition of Vav1 activation by *E. chaffeensis* is also mediated by CD147. To test the requirement for human CD147 in *E. chaffeensis* entry, we previously established a stable knockdown of CD147 in HEK293 cells using lentivirus-based transduction of a small hairpin RNA (shRNA) (1); compared with a small interfering RNA, an shRNA provides sustainable knockdown of target genes with fewer off-target effects (25). HEK293 cells can be readily infected with *E. chaffeensis*, and CD147 gene silencing with shRNA significantly reduces CD147 expression and infection of HEK293 cells with *E. chaffeensis* (1) (Fig. 8A). As endogenous Vav1 levels in HEK293 cells are low, we transfected cells with a Vav1 plasmid that has been used in many Vav1 studies (18, 26) (Fig. 8B). Upon stimulation with PMA, *E. chaffeensis* isolated from THP-1 cells significantly blocked Vav1 activation (tyrosine phosphorylation) in HEK293 cells, compared with HEK293 cells preincubated with medium alone or uninfected THP-1 cell lysate (as negative controls; Fig. 8C). In contrast, Vav1 activation was not affected by *E. chaffeensis* in HEK293 cells transfected with CD147 shRNA, indicating that inhibition of Vav1 activation by *E. chaffeensis* is mediated by CD147.

## DISCUSSION

The present study revealed that *Ehrlichia* uses EtpE to hijack the unique host DNase X-CD147-Vav1 signaling to block Rac 1 activation by host macrophages that generate abundant ROS. CD147 is a type I integral membrane glycoprotein that interacts with multiple mammalian proteins and has many functions, including induction of metalloproteinase activity, regulation of spermatogenesis, expression of the monocarboxylate transporter, and the responsiveness of lymphocytes (27, 28). The present study used CD147^–/–^ BMDM and CD147^–/–^ mice to demonstrate that CD147 is required for infection of macrophages by *E. chaffeensis*. CD147 is not required for ROS generation by macrophages in response to PMA because both WT and CD147^–/–^ BMDM responded to PMA in the same way. However, we found that CD147 mediates the suppression of ROS generation caused by the binding of *E. chaffeensis* or EtpE-C to DNase X. This constitutes the first report of CD147 involvement in regulating ROS production. Interestingly, CD147, Vav1, and activated Rac1 are components of the same downstream pathway in activated T cells, as demonstrated by the finding that overexpression of CD147 interferes with T-cell signaling that is dependent on Vav1 and Rac1 (overexpression of Vav1 or Rac1Q61L, which cannot be regulated by Vav1, disrupts T-cell signaling) (18). However, unlike our results showing the requirement for CD147 in *E. chaffeensis* inhibition of Vav1 activation in response to PMA, overexpression of CD147 in T cells inhibits Vav1 activation (18).

Binding of the Plasmodium falciparum surface protein RH5 to CD147 on the surfaces of erythrocytes is essential for parasite invasion (29). CD147 acts as a functional receptor for measles virus on epithelial cells (30), and CD147 facilitates HIV-1 infection by interacting with virus-associated cyclophilin A (31). Pathogenic Neisseria meningitidis utilizes CD147 as a receptor for vascular colonization (32). CD147 interacts with NOD2, an intracellular receptor for the bacterial cell wall component muramyl dipeptide, and enhances invasion of Listeria monocytogenes, an intracellular bacterial pathogen (33). However, *E. chaffeensis* lacks muramyl dipeptide (34) and does not directly bind CD147, but upon binding of *E. chaffeensis* or EtpE-C to DNase X, CD147 recruitment to the complex is required for *E. chaffeensis* invasion of macrophages (1).

Vav1 can be activated in human neutrophils in response to treatment with the tripeptide formyl-methionyl-leucyl-phenylalanine (35), and *Vav1^–/–^* mouse bone marrow-derived neutrophils were shown to have a 25 to 35% decrease in PMA-induced NADPH oxidase activity compared with WT neutrophils (35), suggesting an important role for Vav1 in NADPH activation. Inhibition of both Rac2 activity and Vav1 activation is a common component of signaling by integrins to inhibit ROS production by neutrophils upon treatment with this tripeptide and C5a, but not by PMA (15). The suppression of ROS generation that is induced by the EtpE-C−DNase X interaction in concert with CD147 seems to differ from integrin-induced suppression of ROS generation in neutrophils, as it blocks PMA-induced ROS generation.

Vav proteins are maintained in an inactive state via an autoregulatory mechanism (26, 36–38). This “closed” structure shifts toward an “open” conformation upon the phosphorylation of tyrosine residues (Y142, Y160, Y174) and C-terminal SH3 (Y836) (26). Several protein tyrosine kinases such as Lck, Src, and Zap70 can activate Vav1 (26). How CD147 mediates *E. chaffeensis*-induced inhibition of tyrosine phosphorylation of Vav1 in response to PMA remains to be investigated.

Anaplasma phagocytophilum, a pathogen closely related to *E. chaffeensis* in the family *Anaplasmataceae*, similarly lacks enzymatic detoxification of ROS, free radical scavenging, repair of ROS-induced damage, the oxidative stress response, and the ability to sequester iron (5, 6); therefore, A. phagocytophilum bacteria isolated from host cells are quite sensitive to ROS (5). A. phagocytophilum preferentially infects granulocytes, but not monocytes, and this bacterium has evolved mechanisms analogous to those of *E. chaffeensis* to block ROS generation upon binding to human neutrophils (39, 40). Specifically, A. phagocytophilum blocks ROS generation in response to Escherichia coli, PMA, formylmethionyl-leucyl-phenylalanine, or the Fc-Oxyburst immune complexes in human and murine neutrophils, but not in monocytes (39, 41). Despite the phylogenetic relatedness of these two bacteria, the absence of DNase X on the surfaces of human neutrophils (7) suggests that A. phagocytophilum uses a distinct signaling pathway to carry out host cell-specific suppression of ROS generation.

The p21-activated kinase 1 protein (PAK1) is an effector of activated Rac1 (42). PAK1 regulates NADPH oxidase activation in human neutrophils (43). It has been reported that the VAV1-Rac1-PAK1 signaling axis regulates phagocyte NADPH oxidase by blocking P47*^phox^* phosphorylation in a stimulus-dependent manner (*N*-formylmethionyl-leucyl-phenylalanine, but not by IgG-immune complex Fcγ receptor [FcγR] signaling) in microglia (42). However, A. phagocytophilum does not block P47*^phox^* phosphorylation in neutrophils (39, 40). Carlyon et al. reported a decrease in Rac2 mRNA level in A. phagocytophilum-infected HL-60 cells (a human leukemia line) and neutrophils at 1 to 2 days postinfection (44), which may be too slow to explain the observed suppression of ROS generation in neutrophils by A. phagocytophilum that occurs within 30 min of interaction (39). Our current study clearly showed that the total Rac1 protein amount in cells did not change, but rather only Rac1 activation was rapidly blocked. Thus, an obvious issue for investigation is whether pathways of the CD147-Vav1-Rac1/2 axis are conserved between *Anaplasma* and *Ehrlichia* species to block ROS generation in their respective host phagocytes.

*E. chaffeensis* is the first example of pathogens that block Rac1 activation to colonize macrophages. Actin polymerization led by Rac/wave activation is utilized for the entry of several intracellular bacteria, including *Listeria*, *Yersinia*, *Salmonella*, and *Chlamydia* into nonphagocytes (45–48). In contrast, the present study clearly showed that *E. chaffeensis* does not activate Rac1, rather actively blocks Rac1 activation. This is in agreement with our previous finding of DNase X and N-WASP-dependent actin polymerization and DNase X-CD147, hnRNPK, N-WASP-dependent *E. chaffeensis* entry into phagocytes and nonphagocytes (1). This unique entry mechanism is probably used in *E. chaffeensis* to avoid NADPH oxidase activation, as Rac-dependent actin polymerization and entry would activate phagocyte NADPH oxidase as well, thus killing *E. chaffeensis*. Taken together, these results indicate that pathways acting upstream of *E. chaffeensis* entry into mammalian cells have dual functions for bacterial entry and suppression of ROS generation, thereby ensuring efficient entry into phagocytes.

## MATERIALS AND METHODS

### Ethics statement and *Bsg^flox/flox-lyz2-Cre^* mice.

All animal experiments were performed in accordance with The Ohio State University Institutional Animal Care and Use Committee guidelines and approved e-protocol. The University program has full continued accreditation by the Association for Assessment and Accreditation of Laboratory Animal Care International (AAALAC-I) under accreditation 000028, dated 9 June 2000, and has a Public Health Services assurance renewal A3261-01, dated 6 February 2019 through 28 February 2023. The program is licensed by the U.S. Department of Agriculture, 1-R-014, and is in full compliance with Animal Welfare Regulations. Myeloid-cell-specific CD147^–/–^ mice:B6J.B6N(129P2)-Bsgtm1.1Riki/Mmmh (Bsg*flox/flox-lyz2-Cre*) were produced at Cyagen Biosciences Inc. (Santa Clara, CA) and deposited into MMRRC (stock number 046273-MU; Citation identifier [ID] RRID:MMRRC_046273-MU). Bsg*flox/flox-lyz2-Cre* and wild-type (WT) C57BL/6 mice (Jackson Laboratory, Bar Harbor, ME) were bred in the animal facilities of The Ohio State University.

### Isolation of mouse BMDM and human PBML.

CD147^–/–^ and WT mice (7 to 12 weeks old) were euthanized, their femurs were removed, and bone marrow-derived macrophages (BMDM) were prepared as described previously (7). Human buffy coat was obtained from the American Red Cross, Columbus, Ohio. Histopaque 10771 (Sigma-Aldrich, St. Louis, MO) was used to isolate peripheral blood mononuclear leukocytes (PBML) as described previously (7).

### Isolation of host cell-free *E. chaffeensis*.

*E. chaffeensis* Arkansas (49) was cultured in the canine macrophage cell line DH82 (50) in Dulbecco’s minimal essential medium (Mediatech, Manassas, VA) or human acute leukemia cell line THP-1 cells (51) in RPMI 1640 medium supplemented with 5% fetal bovine serum (Atlanta Biologicals, Lawrenceville, GA) and 1% l-glutamine (GIBCO, Grand Island, NY) at 37°C in 5% CO_2_ and 95% air in a humidified atmosphere as described previously (51). *E. chaffeensis-*infected cells (∼1 × 10^8^ cells, >90% infected, from two T75 flasks) were harvested by centrifugation at 400 × *g* for 5 min. The pellet was resuspended in culture medium and sonicated on ice for 8 s using a W-380 sonicator (Heat Systems, Newtown, CT) with an output setting of 2. Unbroken cells were removed by centrifugation at 1,000 × *g* for 5 min. The supernatant was collected after additional centrifugation at 1,700 × *g* for 5 min, passed through 2.7-μm and 5.0-μm GD/X nylon filters (Whatman, Florham Park, NJ) to remove cell debris, and centrifuged at 9700 × *g* for 10 min (52). The resulting bacterial pellet was resuspended in Hanks’ balanced salt solution (HBSS; Sigma-Aldrich) supplemented with 2 mg/ml dextrose (Hospira, Lake Forest, IL) (HBSSd). *E. chaffeensis* was quantified based on 16S rRNA gene-based quantitative PCR as described below.

### Isolation of recombinant proteins and coating of latex beads with proteins.

Recombinant EtpE-C and EtpE-N were produced and purified as described previously (19). Carboxylate-modified latex beads (0.5 μm) were coated with rEtpE-C or rEtpE-N solubilized in 6 M urea as described previously (19) at a ratio of 40 ng protein per 5 × 10^6^ beads. Protein-coated beads or uncoated beads were treated with 20 μg/ml polymyxin B sulfate (Sigma-Aldrich) to neutralize possible endotoxin contamination (7, 53).

### Nucleofection of CD147^–/–^ BMDM with a plasmid encoding CD147.

CD147^–/–^ BMDM (5 × 10^6^) were mixed with 5 μg CD147-hemagglutinin (HA) (received from Xose Bustelo, University of Salamanca, Spain) or a GFP-encoded plasmid, 82 μl nucleofection solution, and 18 μl supplement 1 (Amaxa P2 primary cells 4D-Nucleofector X kit L; Lonza, Morristown, NJ) in a 0.2-cm Nucleofector II cuvette. The mixture was subjected to nucleofection using the Amaxa Nucleofector II, program Y-001 (Lonza). Nucleofection was confirmed by Western blotting with anti-HA.

### Luminol-dependent chemiluminescence assay.

To measure ROS, we used a luminol-based chemiluminescence assay in the presence of horseradish peroxidase (HRP) (54). BMDM derived from CD147^–/–^ or WT mice or PBML were cultured in 96-well plates at 5 × 10^5^ cells/well. Luminol (1 mM; Sigma-Aldrich) and 4 U/ml HRP (Sigma-Aldrich) in 150 μl HBSSd were added to each well with incubation at 37°C for 15 min. Each well received 50 μl of rEtpE-C-coated beads, rEtpE-N-coated beads, or uncoated beads at a ratio of 10 beads/cell or additional HBSSd. Alternatively, 50 μl of host cell-free *E. chaffeensis* at ∼100 *E. chaffeensis*/cell or DH82 lysate (derived from the same number of uninfected cells using the same sonication, centrifugation, and filtration methods as for infected cells) was added with incubation for 30 min. After which PMA (200 nM; Sigma-Aldrich) in 50 μl HBSSd was added. HBSSd alone was added to cells as the negative control. For pretreatment with anti-CD147, PBML were seeded in 96-well plates at 5 × 10^5^ cells/well in 150 μl HBSSd in the presence or absence of 10 μg/ml anti-CD147 MEM-M6/6 (low endotoxin, azide free; Abcam, Cambridge, MA), 1 mM luminol, and 4 U/ml HRP with incubation at 37°C for 30 min. EtpE-C- or rEtpE-N-coated beads (∼5 × 10^6^ beads) or HBSSd (50 μl; control) was added to the wells with incubation for an additional 30 min. Then, 200 nM PMA in 50 μl HBSSd was added (HBSSd alone served as the negative control). The plate was continuously read every 20 s with a Synergy HTX multimode reader (Biotek, Winooski, VT) beginning at the point when PMA or HBSSd was added. The area under the curve was measured for 0.5 h and reported as luminescence intensity units as previously described (7).

### Infection of BMDM and mice with *E. chaffeensis*.

A total of 5 × 10^5^ CD147^–/–^ or WT BMDM were cultured in a six-well plate and infected with *E. chaffeensis* at a bacterium-to-cell ratio of 100:1 with incubation for 48 h at 37°C, after which DNA was extracted using the QIAamp DNA minikit (Qiagen, Germantown, MD). Each of five CD147^–/–^ or WT mice were inoculated intraperitoneally with *E. chaffeensis*-infected DH82 cells (>90% infection; 10^6^ cells/mouse). After 4 days, the mice were euthanized, and blood samples were collected. Whole blood was centrifuged, buffy coat was isolated, and DNA was extracted. To quantify *Ehrlichia*, an absolute quantification method was used by creating a standard curve of the *Ehrlichia* 16S rRNA gene cloned into plasmid pUC19 as a standard template (7), and bacterial numbers were determined by 16S rRNA gene copy number with quantitative PCR. PCR was performed in the Mx3000P instrument (Stratagene, Waltham, MA). The value was normalized against mouse glyceraldehyde-3-phosphate dehydrogenase (GAPDH) level using specific primers (50).

### Rac1 activation assay with human monocytes.

Human PBML were suspended in RPMI 1640 medium containing 10% fetal bovine serum, and cell density was adjusted to 5 × 10^6^ PBML/ml and seeded in 10 petri dishes with 3-ml total volume (TPP tissue culture dishes [60 by 15 mm]; Thermo Fisher Scientific, Waltham, MA). PBML were cultured for 5 days. After floating cells were removed, adherent monocytes were incubated with 1.5 ml of *E. chaffeensis* (to give a ratio of 100 bacteria per PBML), DH82 cell lysate, or HBSSd (three wells each). In another set of experiments, adherent monocytes were incubated at 37°C for 30 min with 1.5 ml of rEtpE-C-coated beads or rEtpE-N-coated beads at a ratio of 10 beads/cell, or HBSSd (three wells each). PMA (200 nM; Sigma-Aldrich) in 0.5 ml HBSSd was added, except for two wells for the HBSSd groups, which served as the controls for the non-PMA-stimulated monocytes (one well) and GDP-loaded monocytes (one well) and incubated at 37°C for 5 min. A Rac1 activation assay kit (Cytoskeleton, Denver, CO) was used to detect Rac1-GTP bound to the Rac/Cdc42 (p21) binding domain of PAK1, and total Rac1 was determined with Western blotting. Purified recombinant His-Rac1 (20 μg; Cytoskeleton) was used as a positive control. The protein was transferred to a polyvinylidene difluoride membrane (GE Healthcare, Cincinnati, OH), which was incubated with anti-Rac1 (1:500; Cytoskeleton). After the membrane was rinsed, it was incubated with a secondary antibody (HRP-conjugated goat anti-mouse IgG, diluted 1:1,000; KPL, Gaithersburg, MD). Immunopositive bands were detected with an enhanced chemiluminescence detection method using peroxide solution (Thermo Fisher Scientific, Rockford, IL) and luminol enhancer solution (Thermo Fisher Scientific).

### Vav1 activation assay.

THP-1 cells (2.5 × 10^6^/well) were cultured in wells of a six-well culture plate for 3 days, and the cells were then centrifuged and the culture medium was discarded. For the cell pellet in each well, 1.5 ml of rEtpE-C- or rEtpE-N-coated beads in HBSSd was added to two wells each at a ratio of 10 beads/cell (or HBSSd was added as a control) with incubation at 37°C for 30 min. PMA (200 nM; Sigma-Aldrich) in 0.5 ml HBSSd (or HBSSd alone) was added to each of the duplicate wells with subsequent incubation at 37°C for 5 min. After the cells were washed with ice-cold phosphate-buffered saline (137 mM NaCl, 2.7 mM KCl, 10 mM Na_2_HPO_4_, 2 mM KH_2_PO_4_), the cells were lysed via brief sonication (5 s) in 100 μl radioimmunoprecipitation assay (RIPA) buffer (150 mM NaCl, 25 mM Tris [pH 7.8], 1% [vol/vol] Triton X-100, 0.5% [wt/vol] sodium deoxycholate, 1× protease inhibitor cocktail [EMD Millipore, Billerica, MA], and 1× phosphatase inhibitor cocktail [Thermo Fisher Scientific] per 100 μl in each well). All lysates were gently transferred into cold microcentrifuge tubes and cleared by a 10-min centrifugation at 15,000 × *g* at 4°C. Lysates were incubated for 2 h on a rotator at 4°C with 3 μl of a rabbit antibody to Vav1 (Cell Signaling, Danvers, MA). Protein A-conjugated agarose (20 μl; Cell Signaling) was added to each lysate with overnight incubation at 4°C on a rotator. The resultant immune complexes were washed three times with RIPA buffer and centrifuged at 1,000 × *g* for 30 s at 4°C, and the supernatants were carefully aspirated and discarded. The immune complexes were suspended in 40 μl of 2× Laemmli sample buffer and boiled for 3 min. The protein samples were subjected to sodium dodecyl sulfate-polyacrylamide gel electrophoresis (SDS-PAGE) on a 10% gel. The proteins were transferred to a nitrocellulose membrane (GE Healthcare), which was washed once with Tris-buffered saline (10 mM Tris-HCl [pH 8.0], 150 mM NaCl) and blocked with this buffer containing 0.05% (wt/vol) Tween 20 and 5% bovine serum albumin (Sigma-Aldrich) for 2 h at room temperature. Rabbit anti-Vav1 (1:200; Cell Signaling) or mouse monoclonal anti-phosphotyrosine (PY99; 1:500; Santa Cruz Biotechnology, Dallas, TX) was added with incubation at 4°C overnight. The membrane was incubated with HRP-conjugated goat anti-rabbit IgG (1:1,000; KPL) or HRP-conjugated goat anti-mouse IgG (KPL) in Tris-buffered saline (containing 0.05% Tween 20) for 1 h at room temperature. An enhanced chemiluminescence detection method was used to detect the Vac1 and phosphotyrosine signals. Immunopositive bands were detected with the enhanced chemiluminescence detection method descried above.

### Vav1 activation assay in CD147 shRNA-transformed HEK293 cells.

HEK293 or CD147 shRNA-transformed HEK293 cells (1) (2 × 10^6^ cells) were mixed with 5 μg pC.HA plasmid encoding Vav1 (Addgene, Watertown, MA) in a 0.2-cm cuvette (Bio-Rad, Hercules, CA) and subjected to electroporation using the Gene Pulser Xcell system (Bio-Rad) at 100 V and 1,000 μF. Cells were cultured for 48 h at 37°C, after which *E. chaffeensis* was added at a ratio of 100 bacteria per cell (or THP-1 cell lysate as a negative control) with incubation at 37°C for 30 min. The Vav1 activation assay was performed directly on cell lysate using anti-phospho-Vav1 and Vav1 (Cell Signaling) as described above.

### Statistical analysis.

Experiments were conducted independently at least three times. Statistical analysis was performed with a two-tailed Student’s *t* test. For experiments involving more than two groups, an analysis of variance was performed. For all tests, a *P* value of <0.05 was considered significant. All statistical analyses were performed using Prism 8 software (GraphPad, La Jolla, CA).
